# Lanthanide-Doped KLu_2_F_7_ Nanoparticles with High Upconversion Luminescence Performance: A Comparative Study by Judd-Ofelt Analysis and Energy Transfer Mechanistic Investigation

**DOI:** 10.1038/srep43189

**Published:** 2017-02-23

**Authors:** Dekang Xu, Anming Li, Lu Yao, Hao Lin, Shenghong Yang, Yueli Zhang

**Affiliations:** 1 School of Materials Science and Engineering, Sun Yat-Sen University, Guangzhou 510275, Guangdong, China; 2 School of Physics and Engineering, Sun Yat-sen Univeristy, Guangzhou 510275, Guangdong, China; 3 State Key Laboratory of Crystal Material, Shandong University, Jinan 250100, PR China

## Abstract

The development, design and the performance evaluation of rare-earth doped host materials is important for further optical investigation and industrial applications. Herein, we successfully fabricate KLu_2_F_7_ upconversion nanoparticles (UCNPs) through hydrothermal synthesis by controlling the fluorine-to-lanthanide-ion molar ratio. The structural and morphological results show that the samples are orthorhombic-phase hexagonal-prisms UCNPs, with average side length of 80 nm and average thickness of 110 nm. The reaction time dependent crystal growth experiment suggests that the phase transformation is a thermo-dynamical process and the increasing F^−^/Ln^3+^ ratio favors the formation of the thermo-dynamical stable phase - orthorhombic KLu_2_F_7_ structure. The upconversion luminescence (UCL) spectra display that the orthorhombic KLu_2_F_7_:Yb/Er UCNPs present stronger UCL as much as 280-fold than their cubic counterparts. The UCNPS also display better UCL performance compared with the popular hexagonal-phase NaREF_4_ (RE = Y, Gd). Our mechanistic investigation, including Judd-Ofelt analysis and time decay behaviors, suggests that the lanthanide tetrad clusters structure at sublattice level accounts for the saturated luminescence and highly efficient UCL in KLu_2_F_7_:Yb/Er UCNPs. Our research demonstrates that the orthorhombic KLu_2_F_7_ is a promising host material for UCL and can find potential applications in lasing, photovoltaics and biolabeling techniques.

Lanthanide-doped upconversion nanoparticles (UCNPs) have attracted tremendous attention in diverse fields ranging from three-dimensional (3D) display, solar cells, photocatalysis, and biological labelling due to their advantages of sharp emission bandwidths, long luminescence lifetimes and high color purity^1^,^2^,^3^. To achieve highly efficient upconversion luminescence (UCL), one common strategy is to choose low-phonon-energy hosts (typically fluorides), which can effectively minimize the nonradiative decay rates^4^. Many efforts have been paid to tune the UCL in fluoride systems, among which various doping concentrations of lanthanide ions is usually adopted^5^,^6^. However, appreciable quenching in visible luminescence is experimentally observed for UCNPs with high lanthanide doping levels^7^ (greater than 20% for Yb^3+^, for instance) due to the depletion of excitation energy. Therefore, it is urgent to seek a suitable matrix for the minimization of luminescence quenching. Recently, orthorhombic KYb_2_F_7_ nanocrystals with lanthanide ions arranged in tetrad clusters were found to effectively preserve the excitation and minimize the migration of excitation energy to defects^8^. So far, the study for orthorhombic KYb_2_F_7_ and KLu_2_F_7_ is only presented in the form of glass-ceramics^9^,^10^ or bulk single crystals^11^. Moreover, only several documents reported of the structural and upconversion properties for the nano-sized KLu_2_F_7_^8^,^12^ and KYb_2_F_7_ matrix^13^, respectively.

The assessment of the performance of other UC hosts is very important, which acts as guideline to the preparation and characterization of the product with novel structure. The hexagonal-phase NaYF_4_ has been proved many times to be the highly efficient host for UCL^7^. However, the hexagonal NaYF_4_ usually possesses larger size (much more than 100 nm) in hydrothermal condition. It is of vital importance to seek the highly efficient UC nanocrystals (NCs) with much smaller size. Li *et al*. reported the synthesis of the sub-10 nm monodispersed CaF_2_:Yb, Er NCs and showed the enhanced UC performance compared with cubic-NaYF_4_ counterpart^14^. Since then increasing studies of those alternatives to NaYF_4_ had emerged. For example, hexagonal NaLuF_4_ host, similar to the hexagonal NaYF_4_ counterpart, have been proved to be an excellent host material for UCL by several works^15^,^16^,^17^. ScOF has been proposed as a novel host material for single-band UC generation and high energy transfer efficiency, which is due to the shortest Sc^3+^-Sc^3+^ distance and unique Sc site with specific coordination environment^18^. Therefore, it is significant to fabricate the orthorhombic KLu_2_F_7_ host matrix and theoretically evaluate the UCL performance for further optical investigation.

Herein, we report the facile hydrothermal synthesis of orthorhombic KLu_2_F_7_ nanoparticles with hexagonal shape and systematically study their UC behavior. Excellent UCL performance can be observed in the fabricated products compared with the popular β-NaREF_4_:Yb^3+^, Er^3+^ (RE = Y, Gd) with larger crystal dimension. Our research may enrich the understanding of the synthesis and UCL behavior of KLu_2_F_7_ host matrix.

## Results & Discussion

### Crystal Structures and Morphologies

**Figure 1:**
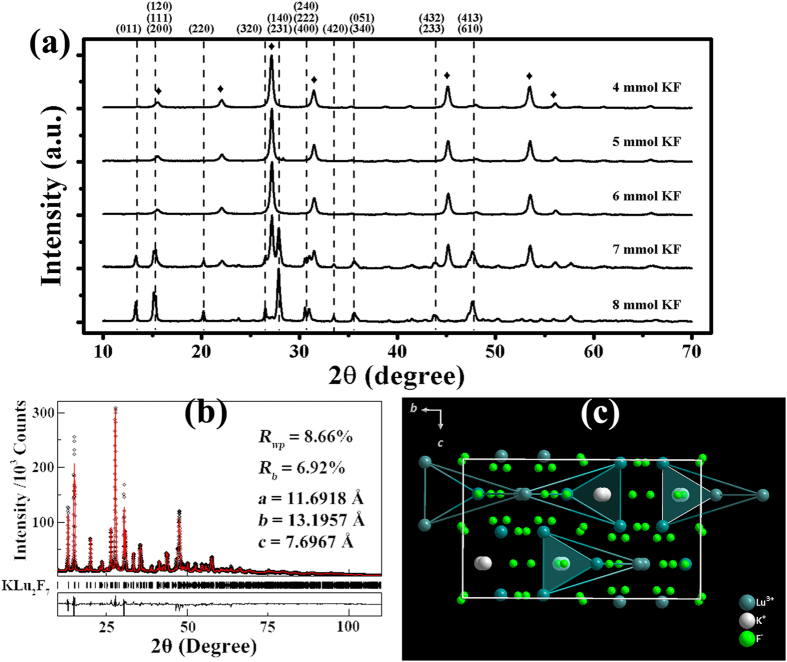
The crystal structure of KLu_2_F_7_:Yb^3+^, Er^3+^ UCNPs. (**a**) XRD patterns of the as-prepared UCNPs with different KF dose. Vertical dashed lines represent standard data of JCPDS 27-0459 for orthorhombic KYb_2_F_7_, and diamond symbols represent standard data of JCPDS 27-0462 for cubic KYb_3_F_10_. (**b**) Rietveld refinement of the orthorhombic KLu_2_F_7_:Yb^3+^, Er^3+^ NCs. The hollow spheres and the red lines stand for experimental and calculated data, respectively. Vertical lines represent the standard orthorhombic structure. The bottom panel displays the residual between the experimental and calculated data. (**c**) Crystal structure of KLu_2_F_7_:Yb^3+^, Er^3+^ NCs according to Rietveld refinement result along [100] projection.

Figure 1 shows the structures of the as-prepared samples. From the XRD patterns, one can observe the phase transition of the samples with the addition of KF. With lesser KF dose, only cubic phase KLu_3_F_10_ is obtained (matches well with JCPDS 27-0462 for cubic KYb_3_F_10_ due to the unavailability of standard pattern of cubic KLu_3_F_10_ and the isostructural character of KLu_3_F_10_ to KYb_3_F_10_, the slight peak shift is due to the smaller Lu^3+^ ionic radius compared with that of Yb^3+^). Then a new structure begins to appear with 7 mmol KF, leading to mixed phases. With the addition of 8 mmol KF, only the pure new structure is observed. Obviously, one can find out that the new structure is almost identical to that of orthorhombic KYb_2_F_7_ (standard data of JCPDS 27-0459) except for the slight shift to larger Bragg angle due to the smaller ionic radii of Lu^3+^ compared with Yb^3+^. Therefore, the observed XRD results can be taken as solid evidence of the formation of orthorhombic KLu_2_F_7_ UCNPs.

To explore the microscopic parameters of the prepared KLu_2_F_7_ structure, the Rietveld refinement based on the least square method is adopted, as revealed in Fig. 1(b). The reliable parameters suggest our sample fits well with orthorhombic structure (space group: Pnam). The lattice parameters of our orthorhombic product (a = 11.6918 Å, b = 13.1957 Å, c = 7.6967 Å) are slightly different from the reported data^19^. The crystal structure, created by Diamond software, is shown in Fig. 1(c), which reveals the tetrad clusters of Lu^3+^ ions at sublattice level, similar with the reported orthorhombic KYb_2_F_7_ structure^8^.

**Figure 2:**
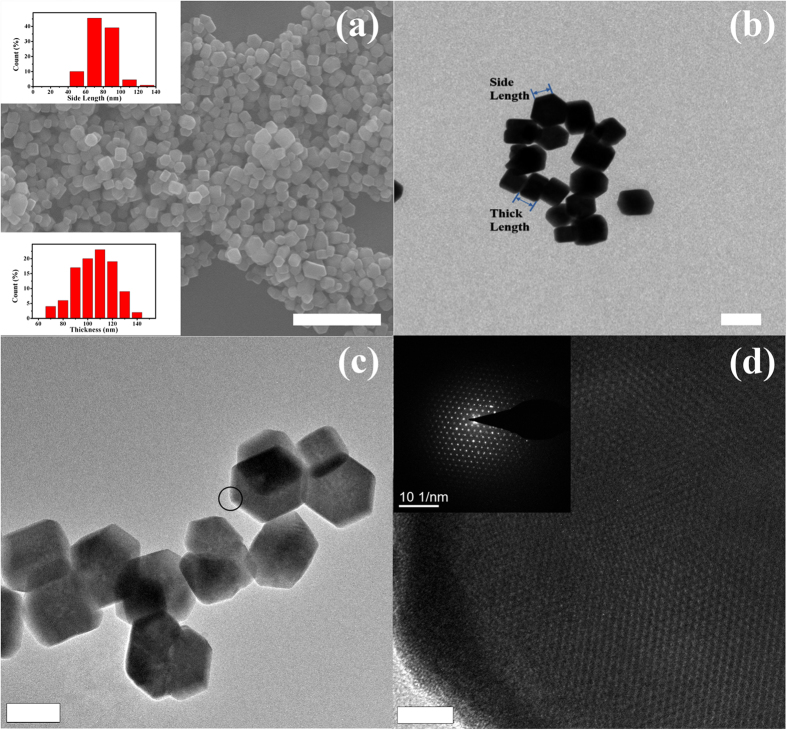
Morphology of the KLu_2_F_7_:Yb^3+^, Er^3+^ UCNPs. (**a**) SEM image of KLu_2_F_7_:Yb^3+^, Er^3+^ UCNPs. Insets show the size histograms of the UCNPs, representing dimension distributions in side length and thickness. (**b**,**c**) TEM image of KLu_2_F_7_:Yb^3+^, Er^3+^ UCNPs. The side length and thickness are marked in (**b**). (**d**) The corresponding HRTEM of the single UCNP noted in (**c**). Inset shows the SAED pattern. Scale bar: (**a**) 1 μm, (**b**,**c**) 100 nm, (**d**) 5 nm.

The corresponding morphologies of all as-prepared samples with different KF dose are shown in Supplementary Fig. S1. From Figure S1(a–e), one can observe that sizes and shapes of the as-prepared UCNPs vary with the change of KF dose. The cubic-phase UCNPs display inhomogeneous and irregular particles with slightly larger dimension ranged from 4 mmol to 6 mmol KF, as can be seen from Supplementary Fig. S1(a–c). With 7 mmol KF, two distinct particle morphologies occur (see Supplementary Figure S1(d)): irregular sub-100-nm particles and uniform hexagonal-shaped particles, which is consistent with the presence of two phases observed from XRD patterns. Figure 2 shows the morphologies of the orthorhombic KLu_2_F_7_:Yb^3+^, Er^3+^ UCNPs. Figure 2(a) shows the pure homogenous hexagonal-prism UCNPs. Insets show the size distribution of the hexagonal shaped UCNPs statistically collected for over 100 particles. The average side length is 70 nm and the average thickness is 100 nm. The representative TEM images (see Fig. 2(b,c)) also verify that our UCNPs are homogenous and dispersible, with hexagonal-shaped particles (the side and thick lengths are marked, respectively). No obvious defects or hollows can be observed, indicating the high crystallinity of our product. Figure 2(d) displays the high resolution TEM (HRTEM) and the corresponding selected area electron diffraction (SAED) pattern of the single UCNP. The obvious lattice fringes and the clear diffraction spots suggest the UCNPs are well crystallized and thus single crystals.

**Figure 3:**
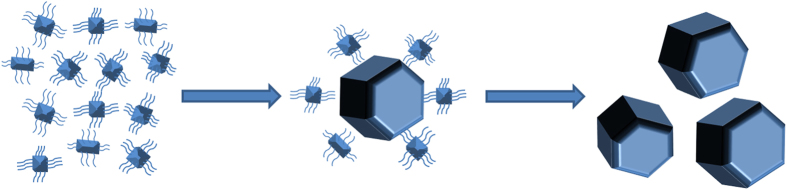
Schematic illustration of the morphological evolution for KLu_2_F_7_ NCs. The smaller polyhedron particles represent the cubic-phase KLu_3_F_10_. The hexagonal-prism particles represent the orthorhombic-phase KLu_2_F_7_. The evolution path is suitable for those situations with increasing KF dose and reaction times.

Many efforts have been made to simultaneously tune the phase and morphology of the UC host materials, such as varying reaction times^20^,^21^ and additives^20^,^22^, and doping with other metal ions^23^,^24^. In this article, changing the ratio of F^−^/Ln^3+^ can also lead to the same effect. Note that cubic KLu_3_F_10_ (F^−^/Ln^3+^ ratio is 3.3) requires a smaller F^−^/Ln^3+^ ratio than that of orthorhombic KLu_2_F_7_ (F^−^/Ln^3+^ ratio is 3.5). During the nucleation process, the particles will be capped with more F^−^ ions in solution with increasing KF dose. We have performed experiments that undergo different reaction times with the other same conditions (see Supplementary Figs S2 and S3). The result shows the phase transformation, from cubic KLu_3_F_10_ to orthorhombic KLu_2_F_7_ (illustrated in Fig. 3), which indicates the process is a thermodynamically-determined process. Therefore, we argue that orthorhombic KLu_2_F_7_ is more thermodynamically stable than cubic KLu_3_F_10_, similar to NaYF_4_ in its hexagonal (β) and cubic (α) forms^23^. According to a previous report^25^, excessive F^−^ could be favorable for phase transformation of NaYF_4_ from α phase to β phase. Similarly, the overload F^−^ content can also lead to phase transformation from cubic KLu_3_F_10_ to orthorhombic KLu_2_F_7_.

### Upconversion performance

**Figure 4:**
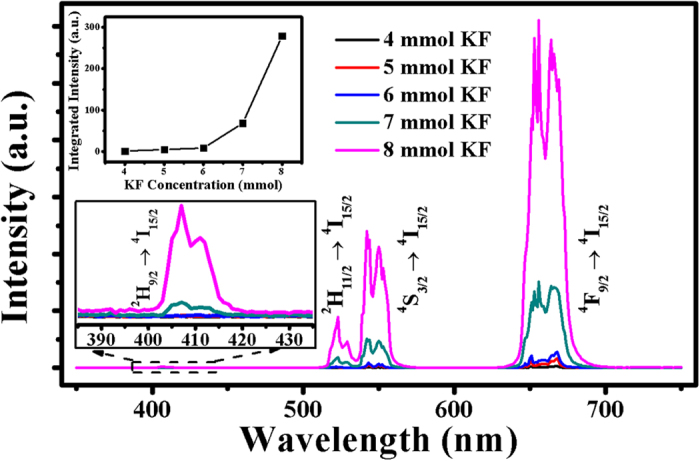
UCL spectra of the as-prepared samples by 980-nm *cw* excitation. Upper inset shows the integrated UCL intensity versus KF concentration. Lower inset shows the enlarged Er^3+^ violet emissions for all samples.

Figure 4 shows the UCL performance of the as-prepared UCNPs by 980-nm *cw* excitation. Two typical emission bands are observed: green emission due to ^2^H_11/2_/^4^S_3/2_ → ^4^I_15/2_ transition and red emission due to ^4^F_9/2_ → ^4^I_15/2_ transition. One can also find the unusual violet emission band, due to ^2^H_9/2_ → ^4^I_15/2_ transition, which, however, appears to be very weak compared to the other two emission bands. This is generally accepted because the violet emission requires more than two photons involved in the UCL process, leading to the lower possibility of energy transition. Nevertheless, the UCL is tremendously enhanced with an elevated level of KF. The total UCL intensity of the orthorhombic-phase UCNPs increases as much as 280 times compared to that of the cubic-phase UCNPs, suggesting the advantage of the orthorhombic structure compared to their cubic-phase counterpart. The extraordinary enhanced violet emission of orthorhombic KLu_2_F_7_ UCNPs compared to cubic KLu_3_F_10_ UCNPs can be attributed to not only the particle dimensions and phase structures but also the confined energy transfer of doped Yb^3+^ clusters within the orthorhombic structure. All the above structural and optical results demonstrate the successful doping of Yb^3+^/Er^3+^ ions into the lower symmetry sites and lanthanide-ion tetrad clusters of the orthorhombic structure.

**Figure 5:**
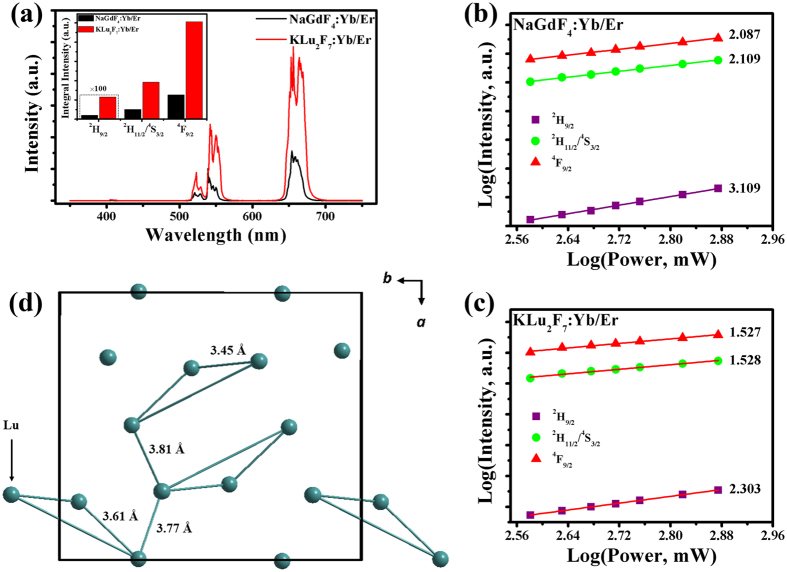
UCL performance of KLu_2_F_7_:Yb^3+^, Er^3+^ NPs by 980-nm *cw* excitation. (**a**) UCL spectra of Yb^3+^, Er^3+^ codoped NaGdF_4_ and KLu_2_F_7_. Inset shows the integral intensity of each emission band for both samples. (**b**,**c**) Log-Log plots of the UCL intensity versus excitation power for violet, green and red emission of Er^3+^ in (**b**) NaGdF_4_:Yb^3+^, Er^3+^ and (**c**) KLu_2_F_7_:Yb^3+^, Er^3+^. (**d**) Schematic representation of the distance between Lu atoms for intra-clusters and inter-clusters along [001] projection based on the results of Rietveld refinement.

To evaluate the UCL performance of the orthorhombic UCNPs, β-NaREF_4_:18%Yb^3+^, 2%Er^3+^ (RE = Y, Gd) is used as reference sample. First, we use NaGdF_4_ as an example (The structure of the compared product was confirmed to be β-NaGdF_4_ by XRD pattern, and the morphology of the product was confirmed to be hexagonal-plate-shape with average dimension size of 1 μm by SEM image, as shown in Supplementary Figures S4 and S5, respectively). As is known to all, β-phase NaREF_4_ is the ideal matrix for efficient UCL^7^. The following results confirm the fact that the orthorhombic-phase host matrix present more excellent UCL performance than the popular β-phase NaREF_4_. From Fig. 5(a), both UC samples exhibit three emission bands, among which the violet emission intensity is much smaller than the other two. One can obviously find that the total luminescence intensity of KLu_2_F_7_:Yb/Er is stronger than that of NaGdF_4_:Yb/Er, which suggests that, in spite of the size effect, the orthorhombic product possesses higher UCL performance than NaGdF_4_:Yb/Er. In addition, we’ve also compared the UCL spectra between KLu_2_F_7_:Yb, Er and NaYF_4_:Yb, Er (The structure and morphology and the UCL spectra are shown in Supplementary Figures S6 and S7, respectively), which also reveals that our product presents excellent UCL performance.

To get deeper insight for the difference of luminescence mechanisms between the above two samples, the power-dependent luminescence intensities for both samples are performed. as depicted in Fig. 5(b,c). A typical two- and three-photon processes are observed for green-/red-emitting and violet-emitting states in NaGdF_4_:Yb/Er sample, respectively. It is comprehensive that the green emission originates from two-photon absorption process, where Er^3+ 4^F_7/2_ manifold is pumped through absorbing one NIR photon by ^4^I_11/2_ manifold after the ground state absorption process triggered by energy transfer from Yb^3+^ to Er^3+^. The red emission can be realized by either of the following channels: 1. ^4^F_7/2_ → ^2^H_11/2_/^4^S_3/2_ → ^4^F_9/2_; 2. ^4^I_11/2_ → ^4^I_13/2_ → ^4^F_9/2_; 3. ^4^F_7/2_ → ^2^H_11/2_/^4^S_3/2_ → ^4^I_13/2_ → ^4^F_9/2_. The former two channels are facilitated by multiphonon relaxation, and the later one is mainly contributed to an energy back transfer (EBT) process. The violet emission is obtained on the basis of the green emission, where another NIR photon is consumed by ^4^F_7/2_ state, followed by the multiphonon relaxation from ^2^G_7/2_ to ^2^H_9/2_. As for the KLu_2_F_7_:Yb/Er sample, the slope values for all three emission bands are smaller than that NaGdF_4_:Yb/Er sample, presenting the more saturated UCL. It becomes reasonable if the depletion of the intermediate states is dominated by energy transfer upconversion (ETU), where the slopes will tend to decrease. This is understandable for the two samples. In NaGdF_4_ host material, the energy migrates in isotropic pathway as in 3D form, which suggests the average distance between Yb^3+^ and Er^3+^ can be expressed in the following formula: *R*_*C*_ = 2(3*V*/(4*πx*_*C*_*N*))^1/3^. *V* is the cell volume. *x*_*c*_ is the critical concentration of Yb^3+^/Er^3+^. *N* is the available site number that the activator can occupy in the cell. From the relevant data, we find that the average separation between Yb^3+^ and Er^3+^ in NaGdF_4_ host material is about 8.96 Å. In KLu_2_F_7_ host material, the special atom clustering structure greatly shortens the distance between Yb^3+^ and Er^3+^, as shown in Fig. 5(d). The average distance of intra-clusters and inter-clusters are about 3.5 Å and 3.8 Å, respectively, which are far smaller than that in NaGdF_4_ host material. The minimized distance between Yb^3+^ and Er^3+^ enables the ETU as dominant depletion mechanism rather than linear decay (LD)^26^, which leads to the saturated luminescence for all emission bands as the slopes tend to decrease^10^,^27^. The ET mechanism for luminescence in KLu_2_F_7_:Yb/Er sample is similar to that in NaGdF_4_:Yb/Er sample, except that the depletion mechanism for the intermediate states is ETU rather than LD, which will be discussed in the subsequent section.

### Judd-Ofelt analysis and energy transfer mechanism

**Figure 6:**
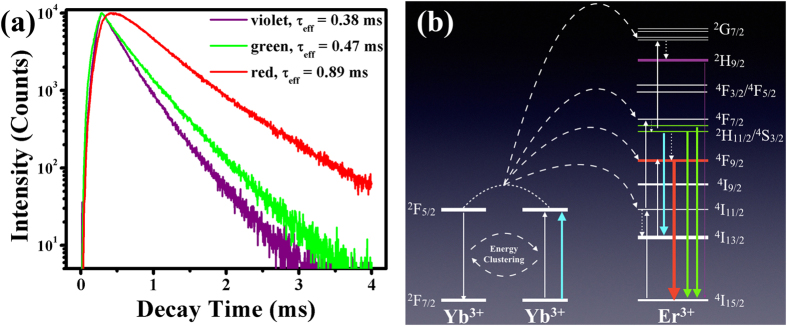
Illustration for ET mechanism in KLu_2_F_7_:Yb^3+^, Er^3+^ UCNPs. (**a**) Luminescence decay curves of three emission bands of Er^3+^ for KLu_2_F_7_:Yb^3+^, Er^3+^ UCNPs under 980-nm pulsed excitation. (**b**) Proposed ET mechanism between Yb^3+^-Er^3+^ in KLu_2_F_7_ host material. Solid arrows, dashed arrows and dotted arrows represent (nonradiative and radiative) transition, ET and multiphonon-relaxation processes, respectively.

To further prove the ET mechanism between Yb^3+^-Er^3+^ in KLu_2_F_7_ UCNPs, the lifetime measurement is performed, as shown in Fig. 6(a). It is obvious that the decay curves do not present linear relationship with the logarithmic intensity, indicating the luminescent process is a complicated energy-transfer process. Therefore, the effective lifetime can be estimated using the following formula instead of the typical exponential decay behavior^1^,^28^: 
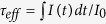
, where *I*_0_ and *I*(*t*) represent the maximum emission intensity and emission intensity at time *t* after the cutoff of the excitation light, respectively. The measured lifetimes for violet (407 nm), green (545 nm) and red emission (656 nm) are 0.38, 0.47 and 0.89 ms, respectively, which are larger than those values for NaGdF_4_:Yb/Er (Supplementary Fig. S8). According to some previous reports^28^,^29^, the experimental transition rate of an excited state (*τ*) involved in an ET process is consisted of all possible radiative and nonradiative transition rates, expressed as: 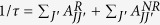. Additionally, the luminescent quantum efficiency (LQE) of a given energy transition is defined as: 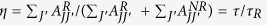. Therefore, in order to obtain the LQE, one has to calculate the spontaneous radiative lifetime, which will be available through Judd-Ofelt (J-O) analysis. In a typical J-O theory, the intensity parameters Ω_*λ*_ are determined by a least-square method combing with the integral absorption coefficients, which is available for rare-earth ions in glasses or solutions. However, when it comes to powder or colloid systems, the absorption coefficients are difficult to obtain due to the uncertain rare-earth ion density and sample thickness. The problem was resolved in a thin-film system by Yang *et al*.^30^, where they used an indefinite constant involved the above two factors and obtained the final J-O parameters by comparing the difference between electric- and magnetic transitions of a given energy level from the prospective of mathematical calculation. Such method can also be extended to the powder or colloid systems. Therefore, we define a constant parameter *K*_*NL*_ (*K*_*NL*_ is a factor including the product of rare-earth ion density and sample thickness) as an unknown quantity. The constant parameter can then be determined by comparing the only electric-dipole transitions with both electric-/magnetic-dipole transitions (As to our case, there is only one energy transition, Er^3+ 4^I_15/2_ → ^4^I_13/2_, that includes both electric- and magnetic-dipole components within the range of lower energies). Once the exact intensity parameters are determined, all the other efficiency parameters such as radiative transition rates, branching ratios and luminescent quantum efficiency can be obtained. Based on the measured absorption spectra of KLu_2_F_7_:Yb/Er and NaGdF_4_:Yb/Er (see Supplementary Figs S8 and S9), the corresponding J-O parameters and predicted efficiency parameters for both samples can be calculated. Related Judd-Ofelt analysis will be processed in the Supplementary Information.

Using the constants given in Supplementary Table S1, one can obtain the parameters such as line strengths, radiative transition rates, branching ratios and radiative lifetimes of the specific manifolds and so on, as shown in Supplementary Tables S2 and S3. We extract and compare the spontaneous transition rates of the corresponding manifolds for the two samples, along with their intensity parameters, as shown in Table 1. The results display following information: 1. The LQE of Er^3+^ violet- and red-emitting manifolds are over 100%, indicating the energies of these manifolds are totally depleted by radiative transition, which means, in other words, luminescence. In contrast, the LQE of Er^3+^ green-emitting manifold are smaller than 100%, suggesting the depletion of ^2^H_11/2_/^4^S_3/2_ manifolds can also be realized by nonradiative process, such as cross-relaxation, multiphonon or ETU. The LQE of Er^3+^ green-emitting manifolds for KLF is much smaller than that for NGF, indicating the depletion for the given manifolds is mainly dominated by nonradiatvie process for KLF; 2. *Ω*_*t*_ generally depends on the covalent bonding and crystal structure. *Ω*_2_ is very susceptible to the asymmetry of RE sites and covalency between RE ions and ligand ions. *Ω*_4_ and *Ω*_6_ are related to the rigidity of the host matrix. The smaller *Ω*_2_ value of KLF suggests the higher degree of symmetry of Er^3+^ sites and dominant covalent bonding between Lu^3+^ and F^−^ ions^31^. Moreover, the ratio between *Ω*_4_ and *Ω*_6_, called spectroscopic quality factor^32^, is much higher in KLF (1.55) than that in NGF (0.54), indicating that KLF can be a more promising laser material than NGF in the visible wavelength range.

From the rising part of the decay curves, one can find that the violet emitting state reaches its maximum intensity as the same time as the green emitting states after absorbing several photons, whilst red emitting state encounters a time delay before it reaches its maximum intensity, implying that the origin of population for the red-emitting manifold is complex. In the past decades, many researches focused on the mechanism of the population of the Er^3+ 4^F_9/2_ red-emitting manifold. Early studies contributed the population of ^4^F_9/2_ manifold to the multiphonon^33^,^34^ and cross-relaxation processes^35^,^36^. Recently, an EBT process involving Er^3+ 2^H_11/2_/^4^S_3/2_ and ^4^I_13/2_ manifolds was proposed and proved to account for the greatly enhanced red emission^26^,^28^,^37^. A new UC mechanism involving the population of ^4^F_9/2_ manifold through an EBT process from high-lying level ^4^G_11/2_ was proposed^38^,^39^. From our previous study^40^, the population of Er^3+^ red-emitting manifold should not be tailored mainly by cross-relaxation or multiphonon processes in Yb^3+^-Er^3+^ system. Therefore, we compare the two disputed EBT processes (see Supplementary Fig. S11) and find out that the main UC mechanism for Er^3+ 4^F_9/2_ population is the EBT process involving Er^3+ 2^H_11/2_/^4^S_3/2_ and ^4^I_13/2_ manifolds (discussed in the Supplementary Information), which is also verified by the above LQE analysis. In a word, the KLF UCNPs present a more saturated luminescence, which is accounted for by the fact that ETU as dominant depletion due to the unique lanthanide-ion tetrad-clusters structure. The above discussion strengthens the viewpoint that the orthorhombic KLu_2_F_7_ can be an efficient host material for UCL.

## Conclusion

KLu_2_F_7_ hexagonal-prism UCNPs are hydrothermally synthesized by controlling the ratio of F^−^/Ln^3+^. The results show the phase transformation from cubic KLu_3_F_10_ to orthorhombic KLu_2_F_7_ is a thermos-dynamical process, and the increasing F^−^/Ln^3+^ ratio favors the formation of thermodynamically stable phase - orthorhombic KLu_2_F_7_. The as-prepared orthorhombic-phase KLu_2_F_7_ UCNPs present much more efficient UCL, which is about 280 times the cubic-phase counterpart. The UCNPs also exhibit better UC emission intensity compared with the known β-NaREF_4_ (RE = Y, Gd) host material. The enhanced UCL is due to the saturated luminescence within the lanthanide tetrad clusters that can well preserve the excitation energy and enable ETU as dominant depletion for intermediate manifolds. Through a modified J-O theory calculation, it is found that KLu_2_F_7_ presents excellent UCL performance and is suitable as lasing materials, rather than NaGdF_4_ host matrix. Our investigation suggests that KLu_2_F_7_ UCNPs can be a good candidate for efficient UCL, and may find potential applications in optoelectronic devices and bioimaging techniques.

## Methods

### Fabrication of UCNPs

The UCNPs (KLu_2_F_7_:Yb^3+^, Er^3+^) were prepared by a facile hydrothermal method. To be specific, a total amount of 1 mmol Ln(NO_3_)_3_ (Ln = 80%Lu, 18%Yb, 2%Er) was added to 10 mL deionized water with agitation. Then 3 mmol dipotassium ethylene diamine tetraacetate (K_2_-EDTA) solution (0.4 M) was added to form a white turbid liquid. The transparent colloid was formed by subsequently adding designated amount of KF, and kept stirred for 30 min before sealed into the autoclave and heated at 200 °C for 12 h. The final products were collected by centrifugation, washed by ethanol and dried at 80 °C overnight.

### Fabrication of the compared sample

#### Preparation of β-NaGdF_4_:18%Yb^3+^, 2%Er^3+^ sample

The compared sample in this article, known as β-NaGdF_4_:18%Yb^3+^, 2%Er^3+^, was prepared with a similar process. Lu^3+^ ions were replaced by Gd^3+^ ions. Citric acid was used instead of K_2_-EDTA solution, and the fluoride source was NaF. The above materials were mixed together and stirred for 30 min. Then the mixture was transferred into the autoclave and dried at 200 °C for 12 h. The final product was collected by centrifugation, washed by ethanol and dried at 80 °C overnight.

#### Preparation of β-NaYF_4_:18%Yb^3+^, 2%Er^3+^ sample

Lu^3+^ ions were replaced by Y^3+^ ions. CTAB was used instead of K_2_-EDTA solution, and the fluoride source was NaF. To obtain sub-micro size particles, 5 ml ethanol was used as solvent. The above materials were mixed together and stirred for 30 min. Then the mixture was transferred into the autoclave and dried at 180 °C for 12 h. The final product was collected by centrifugation, washed by ethanol and dried at 80 °C overnight.

### Characterization

The structural and morphological characterization of the samples were performed on X-ray Diffractometer (Riguaku D-Max 2200 VPC, XRD, Cu Kα radiation), thermal field scanning electron microscope (FEI Quanta 400FEG, SEM, working voltage = 30 kV) and transmittance electron microscope (FEI Tecnai G2 Spirit, TEM, acceleration voltage = 120 kV). UCL spectra were recorded with a Combined Fluorescence Lifetime and Steady-State Spectrometer (Edinburgh FLS920) equipped with a *cw* 980-nm laser diode. The lifetime measurement was performed on a Photoluminescence Spectrometer (Edinburgh FLS980) equipped with a pulsed 980-nm laser diode.

## Additional Information

**How to cite this article:** Xu, D. *et al*. Lanthanide-Doped KLu_2_F_7_ Nanoparticles with High Upconversion Luminescence Performance: A Comparative Study by Judd-Ofelt Analysis and Energy Transfer Mechanistic Investigation. *Sci. Rep.* **7**, 43189; doi: 10.1038/srep43189 (2017).

**Publisher's note:** Springer Nature remains neutral with regard to jurisdictional claims in published maps and institutional affiliations.

## Supplementary Material

Supplementary Information

## Figures and Tables

**Table 1:**
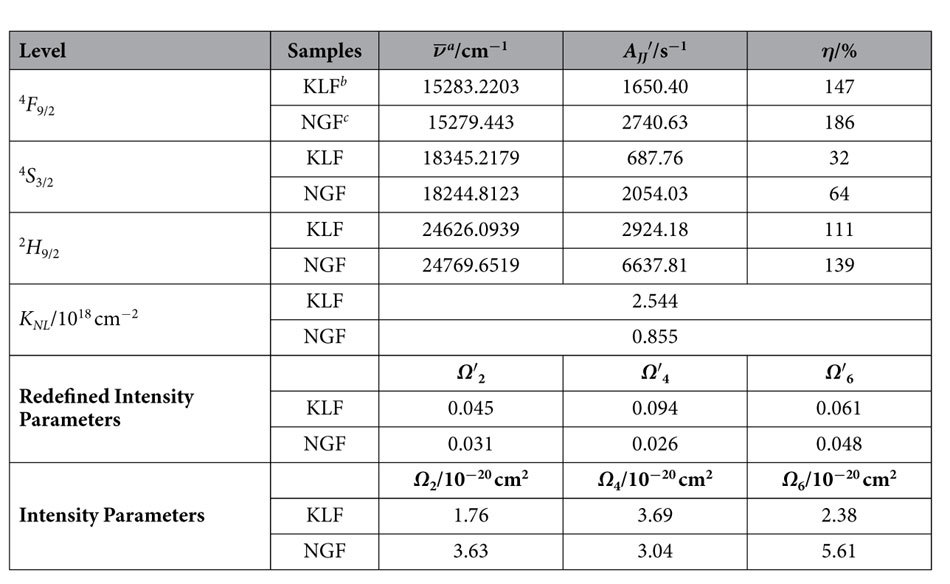
Values of Judd-Ofelt intensity parameters and predicted efficiency parameters for KLu_2_F_7_:Yb/Er and NaGdF_4_:Yb/Er.

^a^The average wavenumbers can be evaluated according to the absorption spectra. The specific calculation is discussed in the supporting information.

^b^KLF represents KLu_2_F_7_:Yb/Er.

^c^NGF represents NaGdF_4_:Yb/Er.
