# Corrigendum: Lanthanide-Doped KLu_2_F_7_ Nanoparticles with High Upconversion Luminescence Performance: A Comparative Study by Judd-Ofelt Analysis and Energy Transfer Mechanistic Investigation

**DOI:** 10.1038/srep46008

**Published:** 2017-04-06

**Authors:** Dekang Xu, Anming Li, Lu Yao, Hao Lin, Shenghong Yang, Yueli Zhang

Scientific Reports 7: Article number: 4318910.1038/srep43189; published online: 02 23 2017; updated: 04 06 2017

In the original version of this Article, there were errors in Affiliation 1 and 2 which were incorrectly listed as ‘School of Materials Science and Engineering, Sun Yat-Sen University, Guangzhou 510275, Guangdong, China’ and ‘School of Physics and Engineering, Sun Yat-sen Univeristy, Guangzhou 510275, Guangdong, China’ respectively.

The correct affiliations are listed below.

Affiliation 1

State Key Laboratory of Optoelectronic Materials and Technologies, School of Materials Science and Engineering, Sun Yat-Sen University, Guangzhou 510275, Guangdong, China.

Affiliation 2

State Key Laboratory of Optoelectronic Materials and Technologies, School of Physics and Engineering, Sun Yat-sen Univeristy, Guangzhou 510275, Guangdong, China.

These errors have now been corrected in the PDF and HTML versions of the Article.

