# Supplementary material for: Lanthanide-Doped KLu2F7 Nanoparticles with High Upconversion Luminescence Performance: A Comparative Study by Judd-Ofelt Analysis and Energy Transfer Mechanistic Investigation
Source: Sci Rep. 2017 Feb 23;7:43189. doi: 10.1038/srep43189 (PMC5322376; doi:10.1038/srep43189)
Supplement: Supplementary Information [file srep43189-s1.pdf]

Lanthanide-Doped KLu_2F_7 Nanoparticles with High Upconversion Luminescence Performance: A Comparative Study by Judd-Ofelt Analysis and Energy Transfer Mechanistic Investigation

Dekang Xu,[1] Anming Li,[2] Lu Yao,[1] Hao Lin,[2] Shenghong Yang,[2] Yueli Zhang*[1,3]

[1] School of Materials Science and Engineering, Sun Yat-Sen University, Guangzhou 510275, Guangdong, China

[2] School of Physics and Engineering, Sun Yat-sen Univeristy, Guangzhou 510275, Guangdong, China

[3]State Key Laboratory of Crystal Material, Shandong University, Jinan 250100, PR China

Corresponding Author: stszyl@mail.sysu.edu.cn

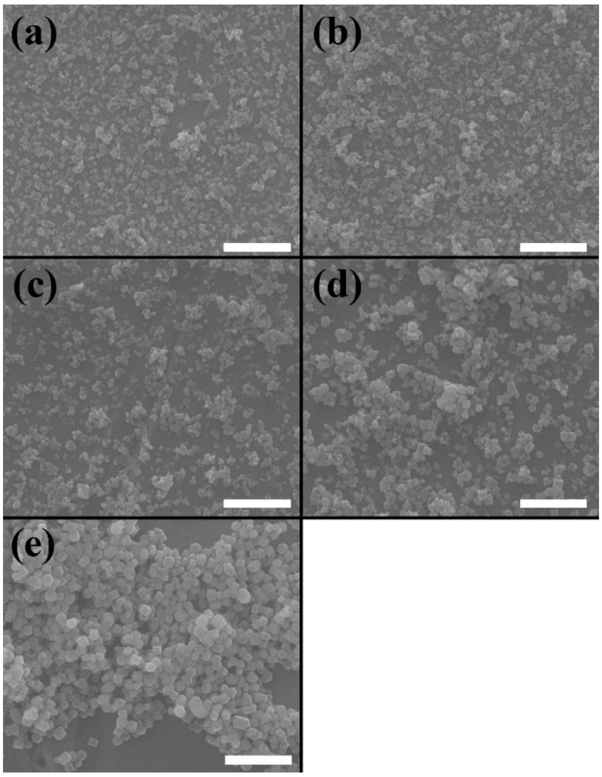

Supplementary Figure S1. (a-e) SEM image of the UCNPs along with different KF dose: (a) 4 mmol, (b) 5 mmol, (c) 6 mmol, (d) 7 mmol, and (e) 8 mmol. Scale bar = 1μm.

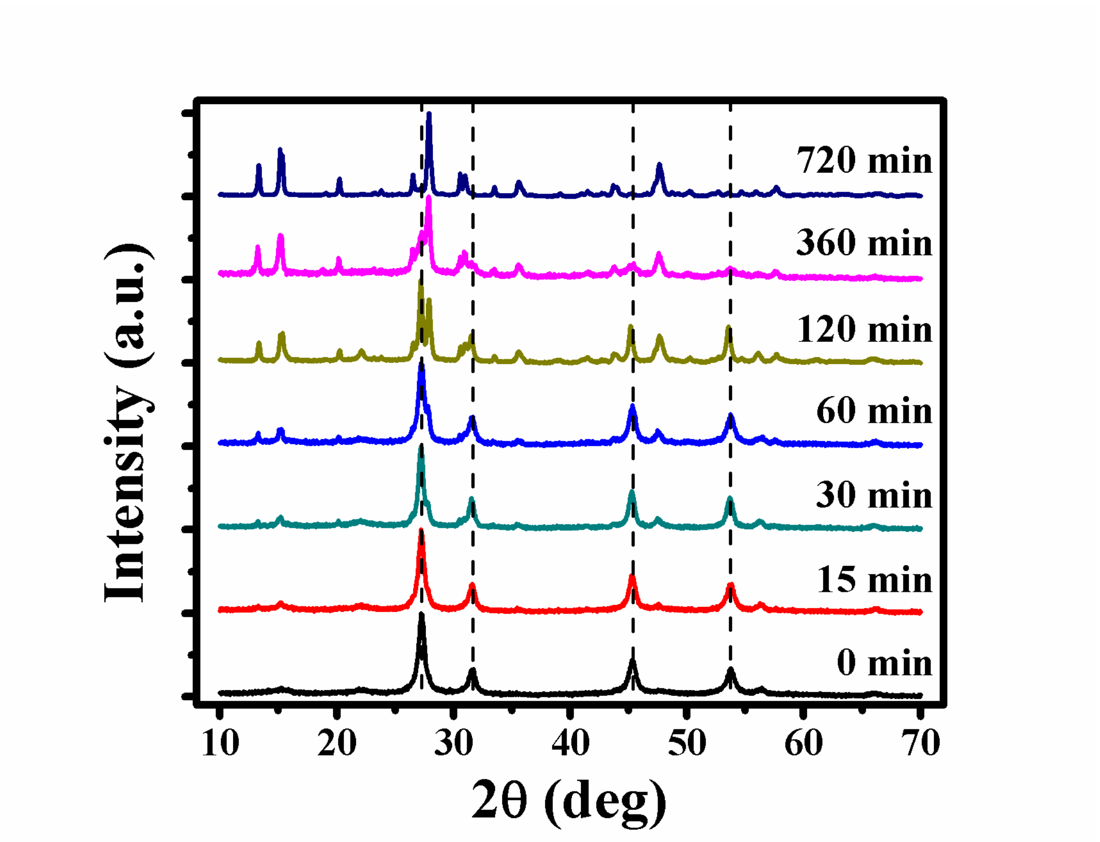

Supplementary Figure S2. XRD patterns of samples with different reaction times for M_F:M_{Ln} = 8:1. The vertical dashed lines represent the cubic-phase KLu_3F_{10} structure. It can be seen clearly that there is a phase transformation along with increasing reaction times (In the initial state, only cubic KLu_3F_{10} can be observed. When the reaction time prolongs to above 30 min, the orthorhombic phase begins to appear, and finally there is only orthorhombic phase after hydrothermally treated for 720 min), revealing the phase transformation process is a thermo-dynamical process and the orthorhombic KLu_2F_7 phase is a thermodynamically stable phase, similar to $NaLnF_4$ in its α and β forms.

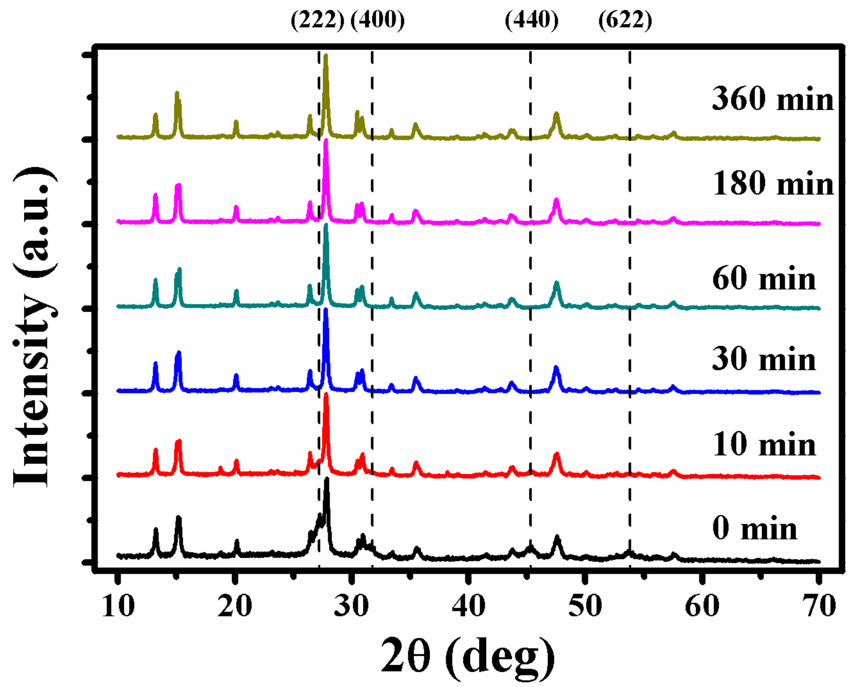

Supplementary Figure S3. XRD patterns of samples with different reaction times for M_F:M_{Ln} = 12:1. The vertical dashed lines represent the cubic-phase KLu_3F_{10} structure. It can be seen clearly that there is also a phase transformation along with increasing reaction times (In the initial state, mixed phases including cubic KLu_3F_{10} and orthorhombic KLu_2F_7 phases coexist. When the reaction time prolongs to above 10 min, the cubic phase begins to disappear, and finally there is only orthorhombic structure), again strengthening the viewpoint that the phase transformation process is a thermo-dynamical process and the orthorhombic KLu_2F_7 phase is a thermodynamically stable phase. Moreover, it is found that the result is different from that with lower M_F:M_{Ln} ratio. With lower M_F:M_{Ln} ratio, the cubic structure is favored in the initial process, whilst the major orthorhombic structure is formed in the beginning with higher M_F:M_{Ln} ratio. The above results demonstrate that more F^- content is in favor of generating the thermodynamically stable phase, similar to the previous reports[1,2].

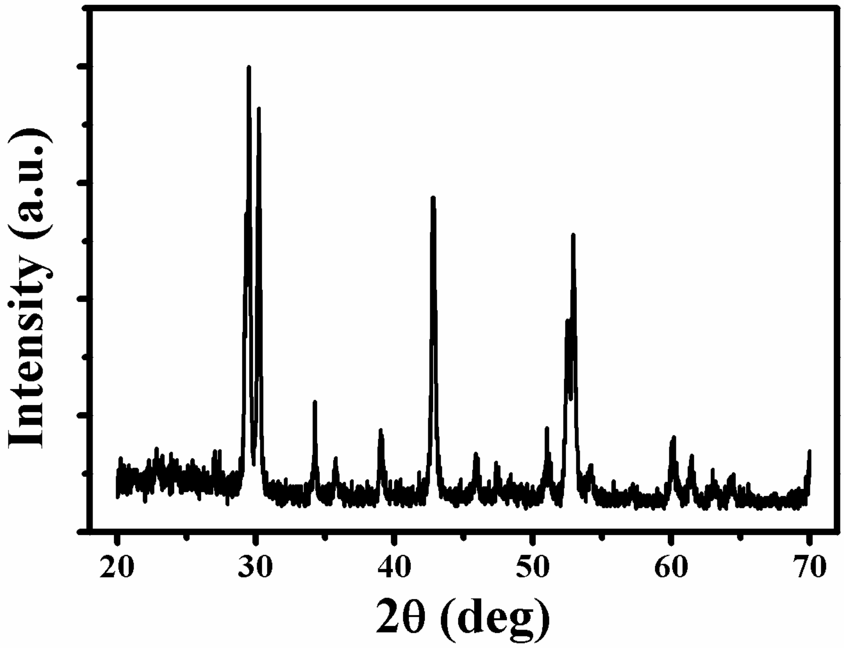

Supplementary Figure S4. XRD pattern of the compared sample $NaGdF_4$:Yb/Er, revealing the typical hexagonal phase.

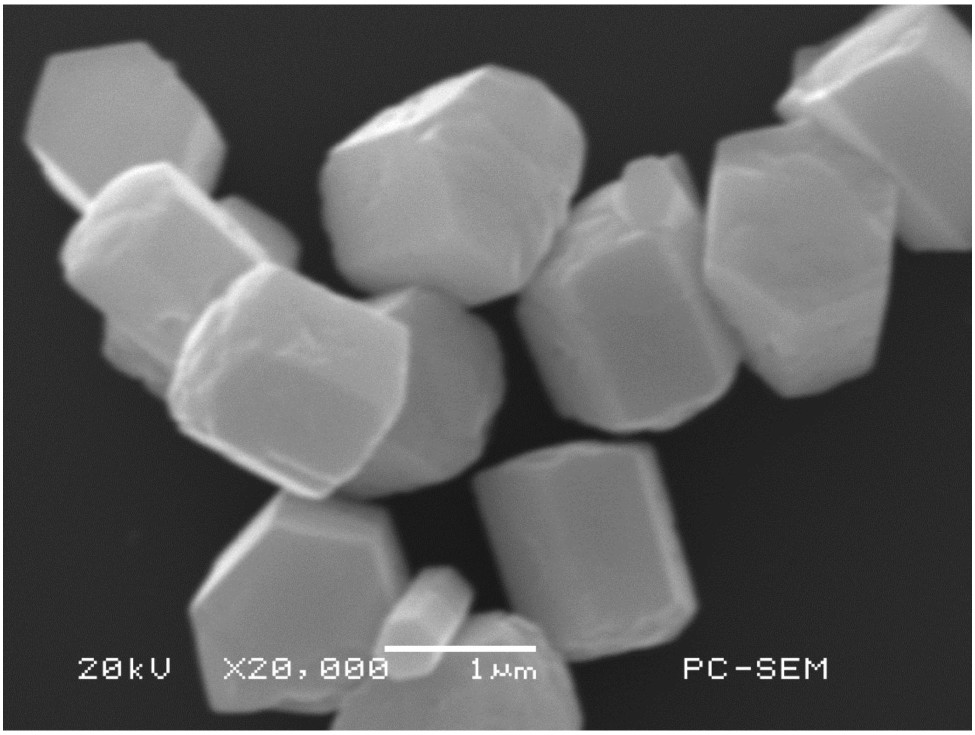

Supplementary Figure S5. SEM image of the compared sample $NaGdF_4$:Yb/Er, showing the hexagonal shaped micro-sized morphology with average dimension about 1 μm.

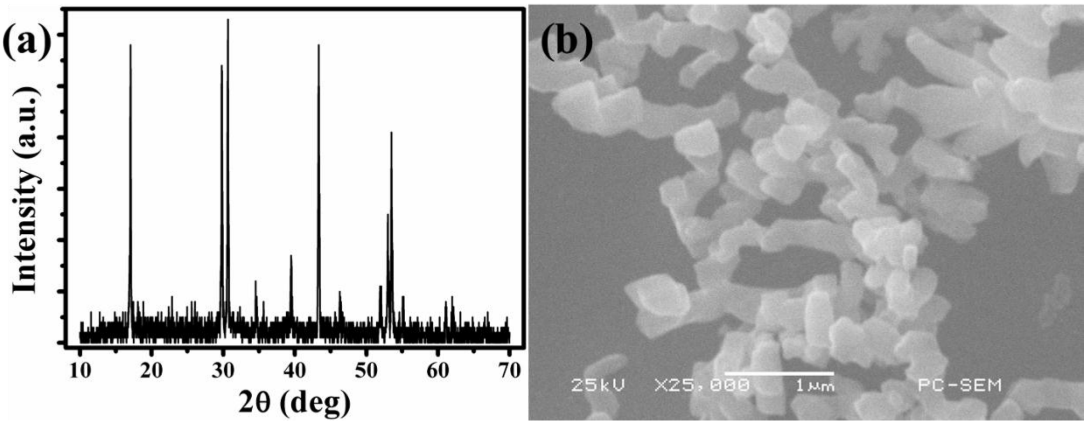

Supplementary Figure S6. XRD pattern and SEM image of $NaYF_4$:18%Yb^{3+},2%Er^{3+}, showing typical hexagonal phase and irregular polyhedron shaped particles with average dimension of about 300 nm.

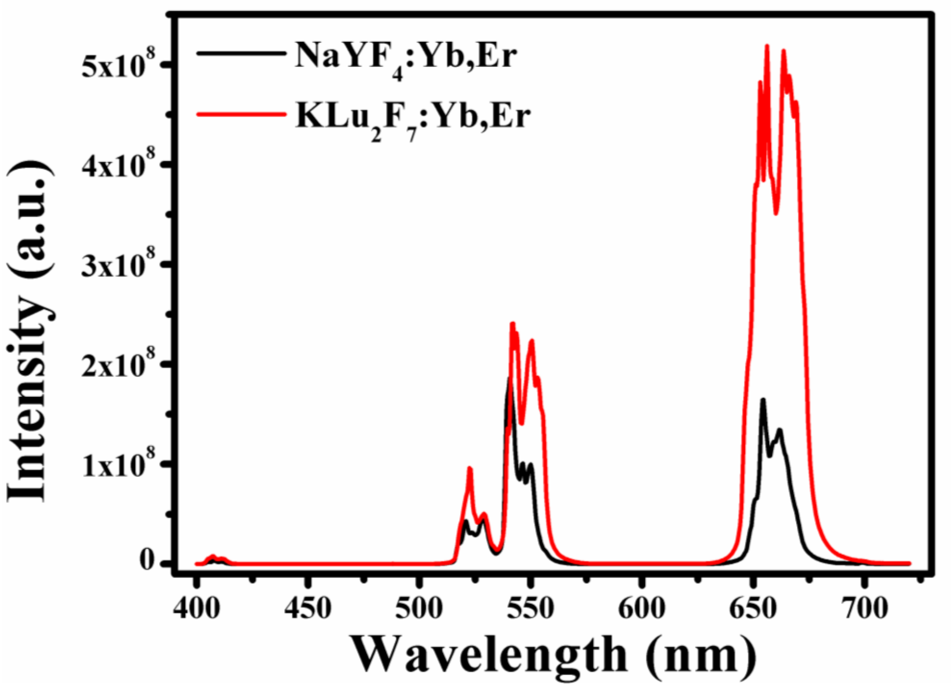

Supplementary Figure S7. UCL performance of KLu_2F_7:Yb,Er versus $NaYF_4$:Yb,Er. The spectra show the stronger UCL intensity of KLu_2F_7:Yb,Er sample compared to $NaYF_4$:Yb,Er, demonstrating directly that our product can be more efficient host material for UCL than the well-known $NaYF_4$.

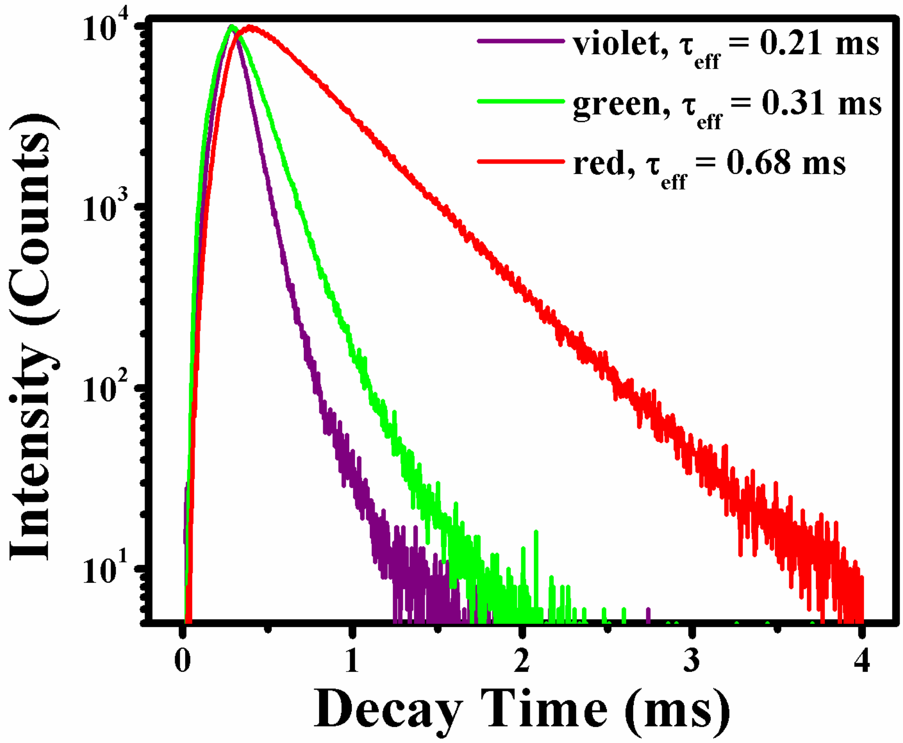

Supplementary Figure S8. Luminescence decay curves of three emission bands of Er^{3+} for β-$NaGdF_4$:Yb/Er under 980-nm pulsed excitation.

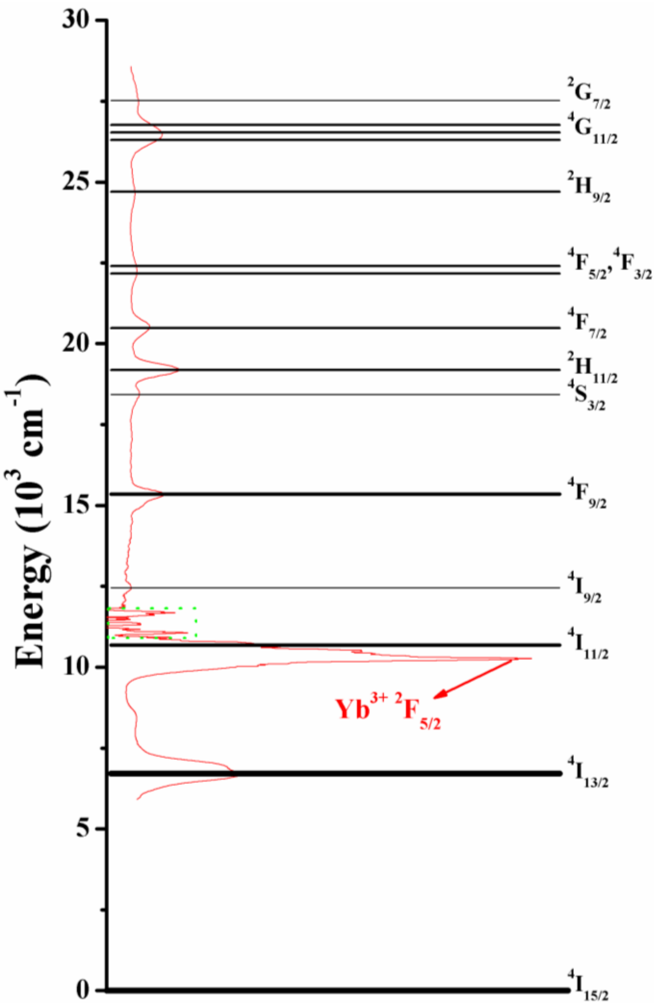

Supplementary Figure S9. Diffuse reflectance spectrum of KLu_2F_7:Yb/Er UCNPs performed on UV3600 UV-vis-NIR spectrometer, superimposed with electronic energy-level for Er^{3+} and Yb^{3+}. The green dotted rectangle region includes some stray peaks, which is due to the error generated by switching detector from visible to NIR. The strongest peak is assigned to Yb^{3+} $^2F_{7/2} \rightarrow {}^2F_{5/2}$ transition due to the large absorption cross-section of Yb^{3+} $^2F_{5/2}$ state.

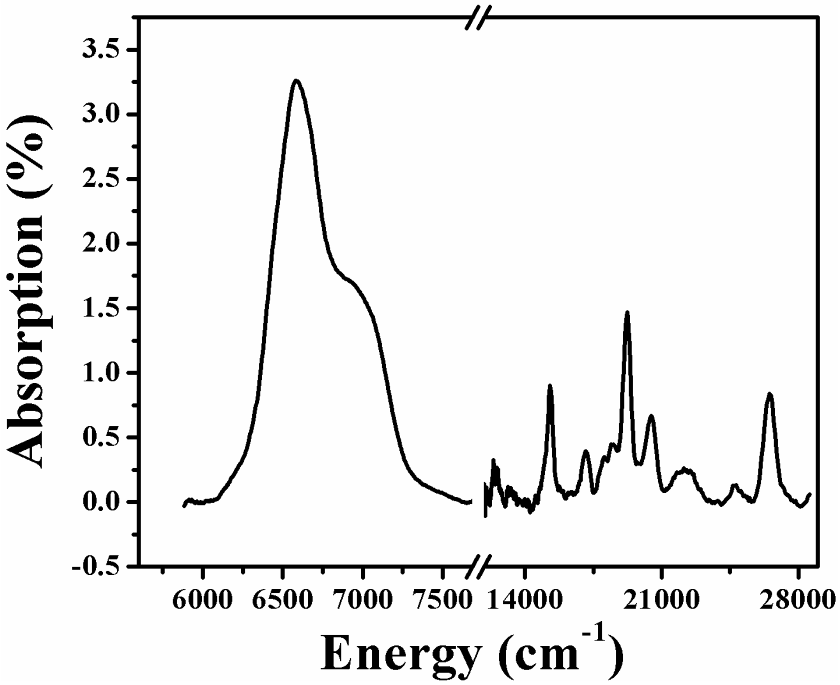

Supplementary Figure S10. Absorption spectra of $NaGdF_4$:Yb/Er, revealing the $^4I_{13/2}$ NIR and visible range of Er^{3+}.

Judd-Ofelt theory analysis

The Judd-Ofelt model is known to calculate the electric- and magnetic-dipole transition spectra line strength for the rare-earth ions embedded in specific host lattices. Herein, we use this theory to calculate the phenomenological intensity parameters of Er^{3+} in Er^{3+}/Yb^{3+} codoped KLu_2F_7 and $NaGdF_4$ host matrix, which further predict the luminescent properties and act as the subsequent proof for the proposed upconversion energy transfer mechanism of Er^{3+} of our samples.

The line strength of electric- and magnetic-dipole transition can be written as follows according to M.J. Weber[3]:

$$S_{ED}(J \to J') = \sum_{\lambda=2,4,6} \Omega_\lambda \left|\langle(SLJ)\middle|U^{(\lambda)}\middle|(S'L'J')\rangle\right|^2$$

$$S_{MD}(J \to J') = \left(\frac{h}{2mc}\right)^2 |\langle(SLJ)|\boldsymbol{L} + 2\boldsymbol{S}|(S'L'J')\rangle|^2$$

The relationship between the absorption spectra and the dipole transition rate is:

$$\int \sigma(\nu)d\nu = \frac{8\pi^3 e^2 \bar{\nu}}{3hc(2J+1)}(\chi_{ED}S_{ED} + \chi_{MD}S_{MD})$$

$\sigma(\nu)$ is the absorption cross-section; χ_{ED} and χ_{MD} are correction factor for $(n^2 + 2)^2/9n$ and n, respectively, where n represents the refractive index of the crystal; $\bar{\nu}$ is mean wavenumbers of the transition related to the absorption spectra, in the form of $\int \nu I(\nu)d\nu / \int I(\nu)d\nu$.

The absorption spectra reflect the relationship between the optical density $D(\nu)$ (or absorbance) and frequency ν, which is $I(\nu) = I_0\exp[-\sigma(\nu)NL]$. The optical density is defined as: $D(\nu) = \int \log(I(\nu)/I_0)\, d\nu$. N is rare-earth ion density in unit volume and L is thickness of the powder sample. However, it is difficult to determine the accurate values of ion density and sample thickness. Hence, it is rational to define a constant parameter $K_{NL} = NL$. According to the above discussion, the total Judd-Ofelt model can be re-written as:

$$2.303\Gamma = \frac{8\pi^3 \bar{\nu}}{3hc(2J+1)}(\chi_{ED}S'_{ED} + \chi_{MD}S'_{MD})$$

$$S'_{ED}(J \to J') = \sum_{\lambda=2,4,6} \Omega'_\lambda \left|\langle(SLJ)\middle|U^{(\lambda)}\middle|(S'L'J')\rangle\right|^2$$

$$S'_{MD}(J \to J') = K_{NL}\left(\frac{h}{2mc}\right)^2 |\langle(SLJ)|\boldsymbol{L} + 2\boldsymbol{S}|(S'L'J')\rangle|^2$$

$\Gamma = \int D(\nu)d\nu$, representing integral optical density for each specific transition; $\Omega'_\lambda = K_{NL}\Omega_\lambda$, representing the redefined intensity parameters. For only electric-dipole transition involved manifolds ($^4F_{9/2}$, $^4S_{3/2}/^2H_{11/2}$, $^4F_{7/2}$, $^2H_{9/2}$ and $^4G_{11/2}$, which are indicated in Supplementary Figure S6), the redefined intensity parameters can be evaluated by a least-square method using the spectra data and the double reduced matrix values referred from M.J. Weber[3]. For electric- and magnetic-dipole transition involved manifolds ($^4I_{13/2}$ only in our situation), we can obtain the exact value for K_{NL} and the final intensity parameters.

The radiative emission rates $A^R_{JJ'}$, branching ratios β and radiative lifetimes τ_R of an excited state J can be evaluated from below:

$$A^R_{JJ'} = \frac{64\pi^4}{3h(2J+1)\bar{\lambda}^3}(\chi'_{ED}S_{ED} + \chi'_{MD}S_{MD})$$

$$\beta = \frac{A^R_{JJ'}}{\sum_{J'} A^R_{JJ'}}$$

$$\tau_R = \frac{1}{\sum_{J'} A_{JJ'}^{R}}$$

where $\chi'_{ED} = n(n^2+2)^2/9$, $\chi'_{MD} = n^3$. Since the total lifetime of an excited state is governed by the combination of probabilities for all possible radiative and nonradiative transitions, which is

$$\frac{1}{\tau} = \sum_{J'} A_{JJ'}^{R} + \sum_{J'} A_{JJ'}^{NR}$$

Therefore, the luminescent quantum efficiency is

$$\eta = \frac{\sum_{J'} A_{JJ'}^{R}}{\sum_{J'} AA_{JJ'}^{R} + \sum_{J'} A_{JJ'}^{NR}} = \frac{\tau}{\tau_R}$$

All the used constants and other parameters are listed in Table S1-S3 for KLu_2F_7:Yb/Er and $NaGdF_4$:Yb/Er, respectively.

Supplementary Table S1. Values of the constants and double reduced matrix for KLu_2F_7:Yb/Er and $NaGdF_4$:Yb/Er.

Levels	Samples	$\bar{\nu}$ / cm^{-1}	U(2)	U(4)	U(6)	Γ / cm^{-1}
$^4I_{13/2}$	KLF	6780.4324	0.0188	0.1176	1.4617	21.4169
	NGF	6735.2397				8.5145
$^4F_{9/2}$	KLF	15283.2203	0	0.5655	0.4651	4.3745
	NGF	15279.4432				2.1203
$^4S_{3/2}$	KLF	18345.2179	0	0	0.2285	1.5499
	NGF	18244.8123				1.9621
$^2H_{11/2}$	KLF	19214.9122	0.7056	0.4109	0.087	6.730
	NGF	19208.472				4.03
$^4F_{7/2}$	KLF	20508.0996	0	0.1467	0.6273	3.4161
	NGF	20461.6716				2.668
$^2H_{9/2}$	KLF	24626.0939	0	0.078	0.170	1.411
	NGF	24769.6519				0.9047
$^4G_{11/2}$	KLF	26481.0796	0.9178	0.5271	0.1197	7.253
	NGF	26518.6654				3.3637
c		2.9979E10 cm/s		***e***	4.8032E-10 esu	
h		6.6261E-27 g.cm^2/s		***m***	9.7094E-28 g	
n	KLF	1.5				
	NGF	1.6				

Supplementary Table S2. Values for the calculated/measured line strength, radiative transition rates, branching ratios and spontaneous emission lifetimes for KLu_2F_7:Yb/Er.

Transition	$\bar{\lambda}$ / nm	S_{cal} / 10^{-20} cm^2	$A_{JJ'}$ / s^{-1}	β	τ_R / μS
$^4I_{13/2} \rightarrow {}^4I_{15/2}$	1475	32.246	201.44	1	4964.26
$^4F_{9/2} \rightarrow {}^4I_{15/2}$	652	3.194	1568	0.95	605.913
$^4I_{13/2}$	1156	0.806	70.945	0.043	
$^4I_{11/2}$	2133	0.772	10.842	6.57E-03	
$^4I_{9/2}$	3505	0.220	0.697	4.22E-04	
$^4S_{3/2} \rightarrow {}^4I_{15/2}$	542	0.544	465.448	0.677	1454
$^4I_{13/2}$	850	0.828	183.836	0.267	
$^4I_{11/2}$	1282	0.201	13.026	0.019	
$^4I_{9/2}$	1676	0.878	25.405	0.037	
$^2H_{9/2} \rightarrow {}^4I_{15/2}$	406	0.692	1409	0.482	341.976
$^4I_{13/2}$	557	1.547	1219	0.417	
$^4I_{11/2}$	715	0.770	286.771	0.098	
$^4I_{9/2}$	823	0.038	9.404	3.22E-03	
$^4F_{9/2}$	1076	0.269	29.371	1.00E-02	

Supplementary Table S3. Values for the calculated/measured line strength, radiative transition rates, branching ratios and spontaneous emission lifetimes for $NaGdF_4$:Yb/Er.

Transition	$\bar{\lambda}$ / nm	S_{cal} / 10^{-20} cm^2	$A_{JJ'}$ / s^{-1}	β	τ_R / μS
$^4I_{13/2} \rightarrow {}^4I_{15/2}$	1485	36.926	485.22	1	2060.92
$^4F_{9/2} \rightarrow {}^4I_{15/2}$	654	4.328	2596	0.947	364.879
$^4I_{13/2}$	1150	1.002	110.367	0.04	
$^4I_{11/2}$	2080	1.735	32.578	0.012	
$^4I_{9/2}$	3450	0.434	1.787	0.0006519	
$^4S_{3/2} \rightarrow {}^4I_{15/2}$	539	1.282	1383	0.674	486.848
$^4I_{13/2}$	836	1.953	564.86	0.275	
$^4I_{11/2}$	1239	0.454	40.311	0.02	
$^4I_{9/2}$	1623	1.658	65.372	0.032	
$^2H_{9/2} \rightarrow {}^4I_{15/2}$	403	1.191	3064	0.462	150.652
$^4I_{13/2}$	549	2.930	2986	0.45	
$^4I_{11/2}$	698	1.152	569.721	0.086	
$^4I_{9/2}$	805	0.058	18.863	0.002842	
$^4F_{9/2}$	1050	0.458	66.692	0.01	

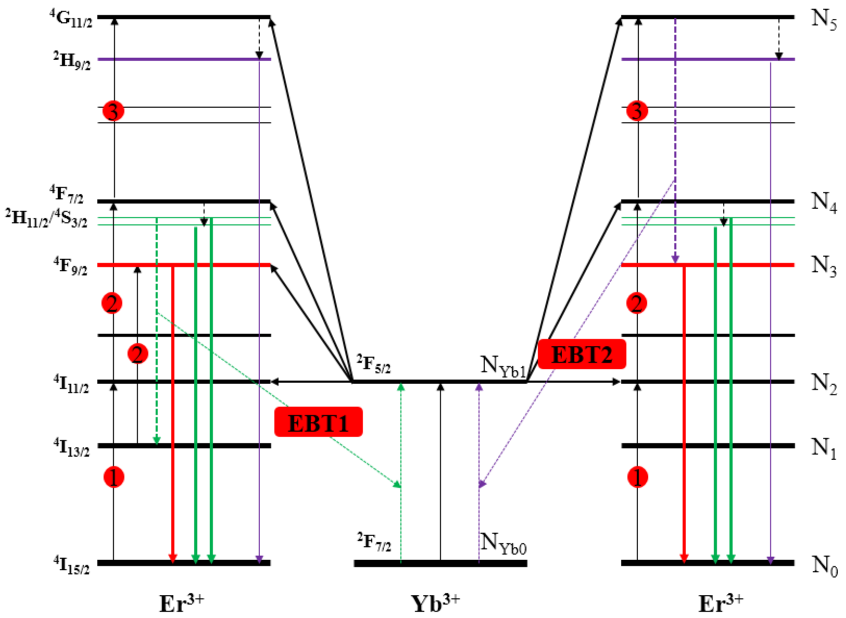

Supplementary Figure S11. Two disputed proposed energy transfer mechanisms interpreting the population of red-emitting manifold. The left panel shows the typical energy back transfer (EBT) process populating Er^{3+} $^4I_{13/2}$ manifold and depopulating Er^{3+} $^2H_{11/2}/^4S_{3/2}$ manifolds. The Er^{3+} $^4F_{9/2}$ manifold will then be populated after absorbing one NIR photon by Er^{3+} $^4I_{13/2}$ manifold. The right panel exhibits the recently found theory[4] that Er^{3+} red-emitting manifold is excited through an EBT process following (Er^{3+}) $^4G_{11/2}$ + (Yb^{3+}) $^2F_{7/2}$ → (Er^{3+}) $^4F_{9/2}$ + (Yb^{3+}) $^2F_{5/2}$.

In the steady-state scenario, the green- and red-emitting population density can be treated and calculated using rate equations. We have used such methods to successfully interpret the energy transfer process between Yb^{3+} and Er^{3+} in several host matrix[5-7]. Generally, the population density of a given transition can be described as:

$$dN_i/dt = \sum_{ij,kl} \left(\omega_{ji,lk}^{ET} N_j N_l - \omega_{ij,kl}^{ET} N_i N_k\right) + \left(\omega_{i+1,i}^{NR} N_{i+1} - \omega_{i,i-1}^{NR} N_i\right) - A_i N_i$$

$\omega_{ij,kl}^{ET}$ is the ET parameters concerned about the donor i to j transition and the acceptor k to l transition. $\omega_{i,i-1}^{NR}$ is the nonradiative MPR rate from the manifold i to the next lower-lying manifold i-1. A_i is the radiative rate of manifold i. Consider the EBT process as main mechanism, all the other cross-relaxation and multiphonon processes can be neglected. Therefore, we have the following equations dealt with the above two proposed energy transfer mechanisms, as shown in Supplementary Table S4.

Of all given transitions, the population density of Yb^{3+} $^2F_{5/2}$ manifold is expressed as:

$$\frac{dN_{Yb1}}{dt} = \sigma\rho N_{Yb0} - \sum_i \omega_i N_i N_{Yb1} - A_{Yb1} N_{Yb1}$$

Under the steady-state pumping condition,

$$N_{Yb1} = \frac{\sigma\rho N_{Yb0}}{\sum_i \omega_i N_i + A_{Yb1}} \propto \rho$$

Supplementary Table S4. Rate equations of the two disputed mechanisms.

Mechanisms	EBT1	EBT2
Equations	$\frac{dN_1}{dt} = \omega_{b1}N_4N_{Yb0} - \omega_1 N_{Yb1}N_1 - A_1N_1$<br>$\frac{dN_2}{dt} = \omega_0 N_{Yb1}N_0 - \omega_2 N_{Yb1}N_2 - A_2N_2$<br>$\frac{dN_3}{dt} = \omega_1 N_{Yb1}N_1 - A_3N_3$<br>$\frac{dN_4}{dt} = \omega_2 N_{Yb1}N_2 - \omega_3 N_{Yb1}N_4 - \omega_{b1}N_{Yb0}N_4 - A_4N_4$<br>$\frac{dN_5}{dt} = \omega_3 N_{Yb1}N_4 - A_5N_5$	$\frac{dN_2}{dt} = \omega_0 N_{Yb1}N_0 - \omega_2 N_{Yb1}N_2 - A_2N_2$<br>$\frac{dN_3}{dt} = \omega_{b2}N_{Yb0}N_5 - A_3N_3$<br>$\frac{dN_4}{dt} = \omega_2 N_{Yb1}N_2 - \omega_3 N_{Yb1}N_4 - A_4N_4$<br>$\frac{dN_5}{dt} = \omega_3 N_{Yb1}N_4 - \omega_{b2}N_{Yb0}N_5 - A_5N_5$
Red / N_3	$\frac{N_0N_{Yb0}N_{Yb1}^3\omega_0\omega_1\omega_2\omega_{b1}}{A_3(A_1+N_{Yb1}\omega_1)(A_2+N_{Yb1}\omega_2)(A_4+N_{Yb1}\omega_3+N_{Yb0}\omega_{b1})}$	$\frac{N_0N_{Yb0}N_{Yb1}^3\omega_0\omega_2\omega_3\omega_{b2}}{A_3(A_4+N_{Yb1}\omega_3)(A_2+N_{Yb1}\omega_2)(A_5+N_{Yb0}\omega_{b2})}$
Green / N_4	$\frac{N_0N_{Yb1}^2\omega_0\omega_2}{(A_2+N_{Yb1}\omega_2)(A_4+N_{Yb1}\omega_3+N_{Yb0}\omega_{b1})}$	$\frac{N_0N_{Yb1}^2\omega_0\omega_2}{(A_2+N_{Yb1}\omega_2)(A_4+N_{Yb1}\omega_3)}$
Violet / N_5	$\frac{N_0N_{Yb1}^3\omega_0\omega_2\omega_3}{A_5(A_2+N_{Yb1}\omega_2)(A_4+N_{Yb1}\omega_3+N_{Yb0}\omega_{b1})}$	$\frac{N_0N_{Yb1}^3\omega_0\omega_2\omega_3}{(A_2+N_{Yb1}\omega_2)(A_4+N_{Yb1}\omega_3)(A_5+N_{Yb0}\omega_{b2})}$
Red-to-Green Ratio / RGR	$\frac{N_{Yb0}N_{Yb1}\omega_1\omega_{b1}}{A_3(A_1+N_{Yb1}\omega_1)}$	$\frac{N_{Yb0}N_{Yb1}\omega_3\omega_{b2}}{A_3(A_5+N_{Yb0}\omega_{b2})}$

From the above results, one can find out that the population density of the violet-, green- and red-emitting manifolds are in similar form in both mechanisms, only with different EBT rates. In our case, ETU is the main depletion of the intermediate states, which means that radiative rates can be neglected compared with UC rate. However, the linear decay is still the main depletion to the luminescent manifolds. Therefore, in EBT1, the population density of the red-emitting state can be expressed as:

$$N_3 = \frac{N_0N_{Yb0}N_{Yb1}\omega_0\omega_{b1}}{A_3(A_4 + N_{Yb0}\omega_{b1})} \propto \rho$$

$$N_4 = \frac{N_0N_{Yb1}\omega_0}{A_4 + N_{Yb0}\omega_{b1}} \propto \rho$$

$$N_5 = \frac{N_0N_{Yb1}^2\omega_0\omega_3}{A_5(A_4 + N_{Yb0}\omega_{b1})} \propto \rho^2$$

$$RGR = \frac{N_{Yb0}\omega_{b1}}{A_3} \propto \rho^0$$

In EBT2,

$$N_3 = \frac{N_0N_{Yb0}N_{Yb1}^2\omega_0\omega_3\omega_{b2}}{A_3A_4(A_5 + N_{Yb0}\omega_{b2})} \propto \rho^2$$

$$N_4 = \frac{N_0N_{Yb1}\omega_0}{A_4} \propto \rho$$

$$N_5 = \frac{N_0N_{Yb1}^2\omega_0\omega_3}{A_4(A_5 + N_{Yb0}\omega_{b2})} \propto \rho^2$$

$$RGR = \frac{N_{Yb0}N_{Yb1}\omega_3\omega_{b1}}{A_3(A_5 + N_{Yb0}\omega_{b2})} \propto \rho^1$$

One can see that the calculated results of EBT1 are corresponding to the experimental data (seen from Figure 5b and 5c). In addition, RGR of EBT1 is independent of pump power, while RGR of EBT2 is proportional to the pump power. Our experimental results (shown in Supplementary Figure S12, demonstrating RGR has nothing to do with excitation power) verify again that the main energy transfer mechanism is EBT1.

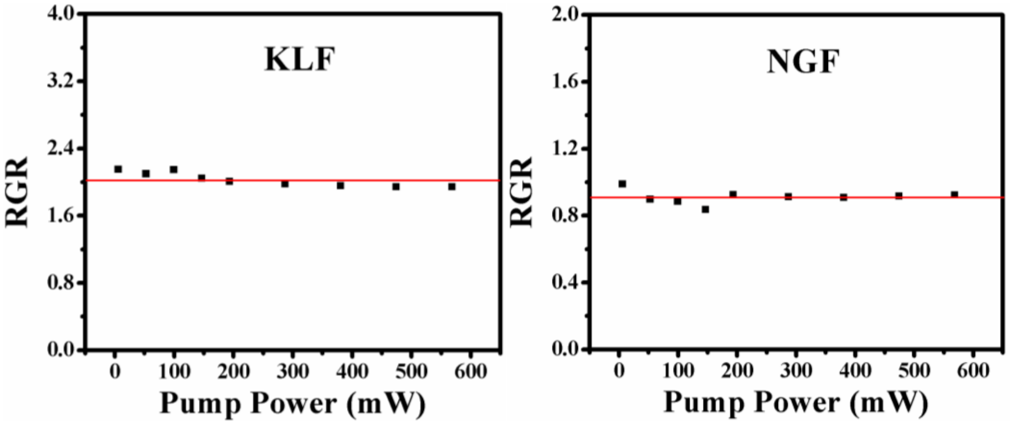

Supplementary Figure S12. Red-to-green ratio versus pump power in KLF and NGF.

1. X. Liang, X. Wang, J. Zhuang, Q. Peng, Y.D. Li, Synthesis of $NaYF_4$ nanocrystals with predictable phase and shap, *Adv. Funct. Mater.*, 2007, **17**, 2757-2765
2. Y.H. Wang, R.X. Cai, Z.H. Liu, Controlled synthesis of $NaYF_4$:Yb,Er nanocrystals with upconversion fluorescence via a facile hydrothermal procedure in aqueous solution, *CrystEngComm*, 2011, **13**, 1772-1774
3. M.J. Weber, Probabilities for radiative and nonradiative decay of Er^{3+} in LaF_3, *Phys. Rev. B*, 1967, **157**, 262-272
4. R.B. Anderson, S.J Smith, P.S. May and M.T. Berry, Revisiting the NIR-to-visible upconversion mechanism in β-$NaYF_4$:Yb^{3+},Er^{3+}, *J. Phys. Chem. Lett.*, 2014, **5**, 36-42
5. D.K. Xu, C.F. Liu, J.W. Yan, S.H. Yang and Y.L. Zhang, Understanding energy transfer mechanisms for tunable emission of Yb^{3+}-Er^{3+} codoped GdF_3 nanoparticles: concentration-dependent luminescence by near-infrared and violet excitation, *J. Phys. Chem. C*, 2015, **119**, 6852-6860
6. A.M. Li, D.K. Xu, Y.L. Zhang, H. Lin, S.H. Yang, Z.Q. Chen and Y.Z. Shao, Upconversion luminescence and energy-transfer mechanism of $NaGd(MoO_4)_2$:Yb^{3+}/Er^{3+} microcrystals, *J. Am. Ceram. Soc.*, 2016, **99**, 1657-1663
7. H. Lin, D.K. Xu, A.M. Li, D.D. Teng, S.H. Yang and Y.L. Zhang, Morphology evolution and pure red upconversion mechanism of β-$NaLuF_4$ crystals, *Sci. Rep.*, 2016, **6**, 28051
